# DataPackageR: Reproducible data preprocessing, standardization and sharing using R/Bioconductor for collaborative data analysis

**DOI:** 10.12688/gatesopenres.12832.2

**Published:** 2018-07-10

**Authors:** Greg Finak, Bryan Mayer, William Fulp, Paul Obrecht, Alicia Sato, Eva Chung, Drienna Holman, Raphael Gottardo

**Affiliations:** 1 Vaccine and Infectious Disease Division, Fred Hutchinson Cancer Research Center, Seattle, WA, 98109, USA; 2 Vaccine Immunology Statistical Center, Fred Hutchinson Cancer Research Center, Seattle, WA, 98109, USA; 3 Statistical Center For HIV AIDS Research and Prevention, Fred Hutchinson Cancer Research Center, Seattle, WA, 98109, USA

**Keywords:** Assay data, Bioconductor, Collaboration, Data science, Package, Reproducibility, Rmarkdown, Rstats, Version control

## Abstract

A central tenet of reproducible research is that scientific results are published along with the underlying data and software code necessary to reproduce and verify the findings. A host of tools and software have been released that facilitate such work-flows and scientific journals have increasingly demanded that code and primary data be made available with publications. There has been little practical advice on implementing reproducible research work-flows for large ’omics’ or systems biology data sets used by teams of analysts working in collaboration. In such instances it is important to ensure all analysts use the same version of a data set for their analyses. Yet, instantiating relational databases and standard operating procedures can be unwieldy, with high "startup" costs and poor adherence to procedures when they deviate substantially from an analyst’s usual work-flow. Ideally a reproducible research work-flow should fit naturally into an individual’s existing work-flow, with minimal disruption. Here, we provide an overview of how we have leveraged popular open source tools, including Bioconductor, Rmarkdown, git version control, R, and specifically R’s package system combined with a new tool *DataPackageR*, to implement a lightweight reproducible research work-flow for preprocessing large data sets, suitable for sharing among small-to-medium sized teams of computational scientists. Our primary contribution is the *DataPackageR* tool, which decouples time-consuming data processing from data analysis while leaving a traceable record of how raw data is processed into analysis-ready data sets. The software ensures packaged data objects are properly documented and performs checksum verification of these along with basic package version management, and importantly, leaves a record of data processing code in the form of package vignettes. Our group has implemented this work-flow to manage, analyze and report on pre-clinical immunological trial data from multi-center, multi-assay studies for the past three years.

## Introduction

A central idea of reproducible research is that results are published along with underlying data and software code necessary to reproduce and verify the findings. Termed a *research compendium*, this idea has received significant attention in the literature ^1^,^5^.

Many software tools have since been developed to facilitate reproducible data analytics, and scientific journals have increasingly demanded that code and primary data be made publicly available with scientific publications ^2^,^6^,^27^. Tools like git and Github, figshare, and Rmarkdown are increasingly used by researchers to make code, figures, and data open, accessible and reproducible. Nonetheless, in the life sciences, practicing reproducible research with large data sets and complex processing pipelines continues to be challenging.

Data preprocessing, quality control (QC), data standardization, analysis, and reporting are tightly coupled in most discussions of reproducible research, and indeed, literate programming frameworks such as Sweave and Rmarkdown are designed around the idea that code, data, and research results are tightly integrated ^2^,^25^. Tools like Docker, a software container that virtualizes an operating system environment for distribution, have been used to ensure consistent versions of software and other dependencies are used for reproducible data analysis ^16^. The use of R in combination with other publicly available tools has been proposed in the past to build reproducible research compendia ^3^,^28^,^29^. Many existing tools already implement such ideas. The *workflowr* package provides mechanisms to turn a data analysis project into a version-controlled, documented, website presenting the results. The *drake* package ^30^ is a general purpose work-flow manager that implements analytic "plans", caching of intermediate data objects, and provides scalability, and provides tangible evidence of reproducibility by detecting when code, data and results are in sync.

However, tight coupling of preprocessing and analysis can be challenging for teams analyzing and integrating large volumes of diverse data, where different individuals in the team have different areas of expertise and may be responsible for processing different data sets from a larger study. These challenges are compounded when a processing pipeline is split across multiple teams. A primary problem in data science is the programmatic integration of software tools with dynamic data sources.

Here, we argue that data processing, QC, and analysis can be treated as modular components in a reproducible research pipeline. For some data types, it is already common practice to factor out the processing and QC from the data analysis. For rnaseq data, for example, it is clearly impractical and time consuming to re-run monolithic code that performs alignment, QC, gene expression quantification, and analysis each time the downstream analysis is changed. Our goal is to ensure that downstream data analysis maintains dependencies on upstream raw data and processing but that the processed data can be efficiently distributed to users in an independent manner and updated when there are changes.

Here, we present how the Vaccine Immunology Statistical Center (VISC) at the Fred Hutchinson Cancer Research Center has addressed this problem and implemented a reproducible research work-flow that scales to medium-sized collaborative teams by leveraging free and open source tools, including R, Bioconductor and git ^22^,^31^.

## Methods

### Operation

In order to use *DataPackageR* an R installation (≥3.5.0) is required. Associated dependencies are listed in the package’s DESCRIPTION file and are automatically installed when using the *install_github()* API from the *devtools* package. There are no minimum memory, CPU, or storage requirements apart from what is necessary to perform data processing, which varies on a case–by-case basis.

### Implementation

Our work-flow is built around the *DataPackageR* R package, which provides a framework for decoupling data preprocessing from data analysis, while maintaining traceability and data provenance ^18^.

*DataPackageR* builds upon the features already provided by the R package system. R packages provide a convenient mechanism for including documentation as part of the built-in help system, as well as long-form vignettes, and version information and distribution of the entire package. Importantly, R packages often include data stored as R objects, and some packages, particularly under BioConductor, are devoted solely to the distribution of data sets ^22^. The accepted mechanism for such distribution is to store R objects as rda files in the data directory of the package source tree and to store the source code used to produce those data sets in data-raw. The *devtools* package provides some mechanisms to process the source code into stored data objects ^32^.

Data processing code provided by the user (in the form of Rmd files preferably, and R files optionally) is run and the results are automatically included as package vignettes, with output data sets (specified by the user) included as data objects in the package. Additional languages such as *bash* and *python* scripts are supported via rmarkdown’s multi-language code chunk support. Notably, this process, while apparently mirroring much of the existing R package build process, is disjointed from it, thereby allowing the decoupling of computationally long or expensive data processing from routine package building and installation. This allows *DataPackageR* to decouple data munging and tidying from data analysis while maintaining data provenance in the form of a vignette in the final package where the end-user can view and track how individual data sets have been processed. This is particularly useful for large or complex data that involve extensive preprocessing of primary or raw data (e.g. alignment of fastq files for rnaseq or gating of fcm data), and where computation may be prohibitively long or involve software dependencies not immediately available to the end-user.

*DataPackageR* implements these features on top of a variety of *tidyverse* tools including *devtools, roxygen2, rmarkdown, utils, yaml, purrr*. The complete list of package dependencies is in the package DESCRIPTION file.

### Package structure

To construct a data package using *DataPackageR*, the user invokes the datapackage.skeleton() API, which behaves like R’s *package.skeleton()*, creating the necessary directory structure with some modifications. A listing of the structure of *DataPackageR* skeleton package directory, with other associated files is shown below:



                        
package root
|--- datapackager.yml # Configuration file controlling
|                     # the package build process.
|--- DESCRIPTION # Adds a DataVersion  
|                # string to version the 
|                # data set.
|--- NAMESPACE
|--- DATADIGEST # Stores an MD5 hash of each 
|               # data object in the package.
|--- R
|--- Read-and-delete-me.txt    # Further instructions  
|                              # on building the package.
|--- data             # Holds processed, analysis-ready data objects.
|--- data-raw         # User code for data  
|                     # processing is placed here by
|                     # datapackage.skeleton().
|--- documentation.R # Auto generated roxygen documentation
|                    # for data set objects.
|--- inst
| |___ extdata # (small) raw data files.
| |___ doc   # Processed vignettes are moved here.
|            # Data processing code is accessible in the
|            # final package via the vignette() API.
|--- vignettes # Scripts in data-raw
|              # are processed into vignettes.
|___ man # Autogenerated documentation is processed 
         # into rd files. 
                    


The datapackage_skeleton API takes several new arguments apart from the package *name*. First, *code_files* takes a vector of paths to *Rmd* or *R* scripts that perform the data processing. These are moved into the data-raw directory The argument *r_object_names* takes a vector of quoted R object names. These are objects that are to be stored in the final package and it is expected that they are created by the code in the *R* or *Rmd* files. These can be *tidy* data tables, or arbitrary R objects and data structures (e.g. *S4* objects) that will be consumed by the package end-user. Information about the processing scripts and data objects is stored in a configuration file named datapackager.yml in the package root directory and only used by the package build process. The scripts may read raw data from any location, but generally the package maintainer should place it in inst/extdata if file size is not prohibitive for distribution.

### The build_package API

Once code and data are in place, the build_package() API invokes the build process. This API is the only way to invoke the execution of code in data-raw to produce data sets stored in data. It is not invoked through R’s standard R CMD build or R CMD INSTALL APIs, thereby decoupling long and computationally intensive processing from the standard build process invoked by end-users. Upon invocation of build_package() the *R* and *Rmd* files specified in datapackager.yml will be compiled into *package vignettes* and moved into the inst/doc directory, data objects will be created and moved into data, data objects will be version tagged with their *checksum* and recorded in the *DATADIGEST* file in the package root, and a *roxygen* markup skeleton will be created for each data object in the package.

### YAML configuration

The datapackager.yml configuration file in the package root controls the build process by specifying which R and Rmd files should be processed and which named R objects are expected to be included as data sets in the package. The render_root property points to a directory where R and Rmd files will be processed, allowing multi-script pipelines to share file system artifacts. The datapackage_object_read() API also allows downstream scripts to read data objects created by earlier scripts. The listing below shows the structure of the YAML configuration file used by *DataPackageR* to control compilation and inclusion of data objects in the package:



                        
  configuration:
  files:                             # files property lists 
    process_dataset_one.Rmd:         # R or Rmd code files 
      name: process_dataset_one.Rmd  # Each file has a name
      enabled: yes                   # The enabled property specifies
                                     # if the file should be processed
    process_dataset_two.Rmd:
      name: process_dataset_two.Rmd
      enabled: yes
  objects:                           # A list of the data objects created
  - dataset_one                      # by processing the files. 
  - dataset_two
  - dataset_three
render_root:                         # root directory where scripts are 
  tmp: ‘298918’                      # render()ed for multi-script   
                                     # pipelines
                    


The API for interacting with the YAML configuration file is outlined in Table 1.

**Table 1.** The API for interacting with a YAML config file used by DataPackageR allows the user to add and remove data objects and code files, toggle compilation of files, and read and write the configuration to the data package.

API call	Property	return value
yml_add_files()	file	config object
yml_remove_files()	file	config object
yml_add_objects()	objects	config object
yml_remove_objects()	objects	config object
yml_find()	config file	config object
yml_write()	config file	null
yml_enable_compile()	enable	config object
yml_disable_compile()	enable	config object

### Accessing raw data from scripts

Users can access the package root and inst/extdata directories from within R and Rmd scripts in a portable manner using the project_path() and project_extdata_path() APIs. Raw data stored outside the package root should be accessed relative to these locations.

### Dataset versioning

**Figure 1.**
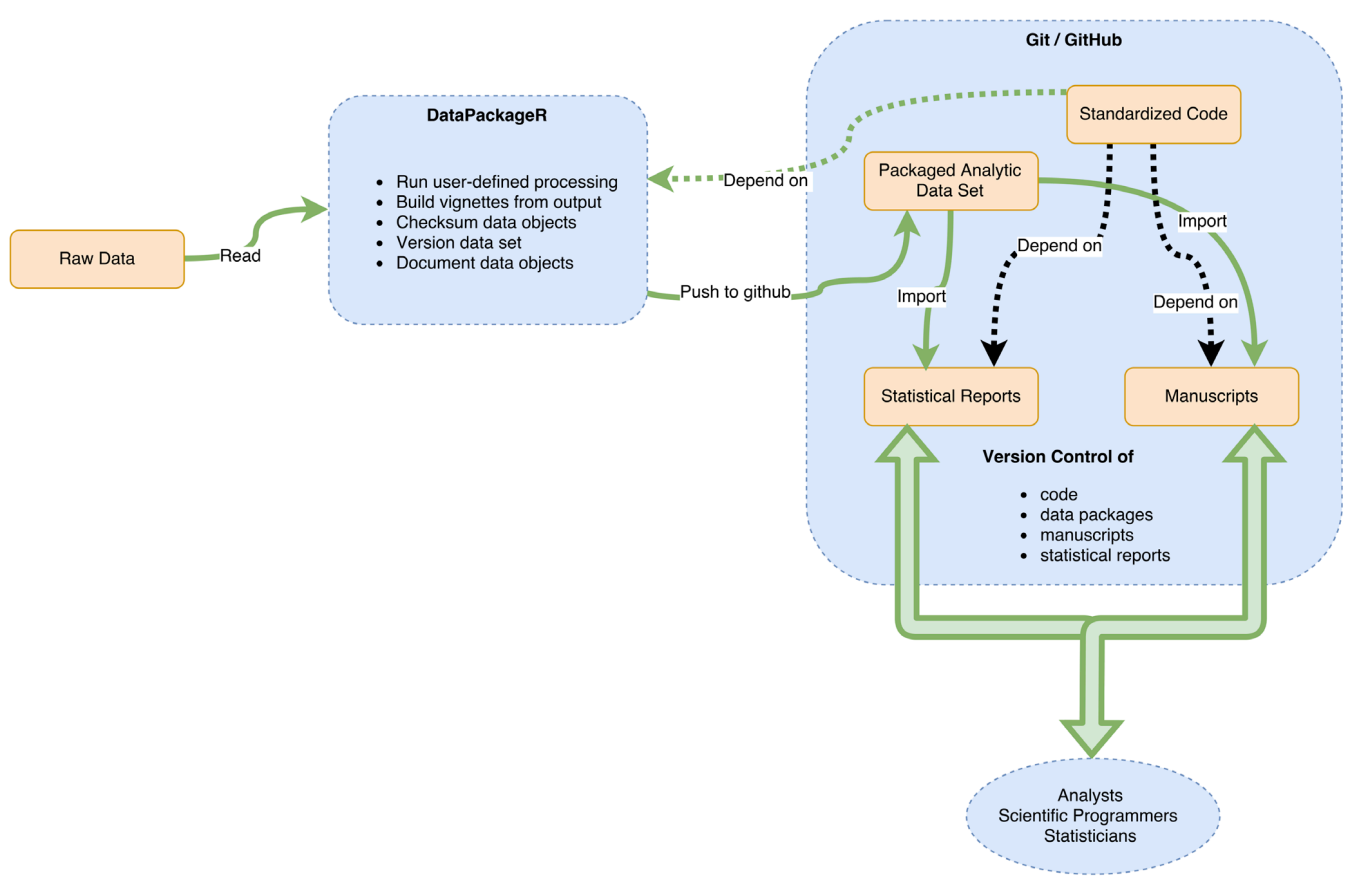
A schematic overview of the components in our reproducible data packaging work-flow that decouples data processing from data analysis.

During the build, the DATADIGEST file is auto-generated. This file contains an md5 hash of each data object stored in the package as well as an overall data set version string. These hashes are checked when the package is rebuilt; if they do not match, it indicates the format of the processed data has changed (either because the primary data has changed, or because the processing code has changed to update the data set). In these cases, the *DATADIGEST* for the changed object is updated and the minor version of the DataVersion string in the DESCRIPTION file is automatically incremented. The DataVersion for a package can be checked by the data_version() and assert_data_version() APIs, allowing end-users to produce reports based on the expected version of a data set ( Figure 1).

### Data documentation

*DataPackageR* ensures that documentation is available for each data object included in a package by automatically creating a *roxygen* markup stub for each object that can then be filled in by the user. Undocumented objects are explicitly excluded from the final package.

Packages can be readily distributed in source or tarball form (together with the processed data sets under data/ and raw data sets under inst/extdata). Within VISC we leverage *git* and *github* to provide version control of data package source code. By leveraging *DataPackageR*, the data processing is decoupled from the usual build process and does not need to be run by the end-user each time a package is downloaded and installed. Documentation in the form of *Rd* files, one for each data object in the package, as well as *html vignettes* describing the data processing, are included in the final package. These describe the data sets as well as how data was transformed, filtered, and otherwise processed from its raw state.

## Use cases

*DataPackageR* was developed as a lightweight alternative to existing reproducible work-flow tools (e.g. *Galaxy* ^33^), or to fully fledged database solutions that are often beyond the scope of most short-term projects. *DataPackageR* plugs easily into any existing R-based data analysis work-flow, since existing data processing code needs only to be formatted into Rmarkdown (ideally). It is particularly suited for long-running or complex data processing tasks, or tasks that depend on large data sets that may not be available to the end user (e.g. FASTQ alignment or raw flow cytometry data processing). Such tasks do not fit well into the standard R CMD build paradigm, for example either as vignettes or *.R* files under /data since these would be invoked each time an end user builds a package from source. We desire, however, to maintain a link between the processed data sets and the processing code that generates them. We note that *DataPackageR* is distinct from other reproducible research frameworks such as *workflowr* or *drake* ^30^, in that it is designed to *reproducibly prepare data for analysis*, using an *existing code base*, with little additional effort. The product of *DataPackageR* is nothing more than an R package that can be used by anyone. The resulting data packages are meant to be shared, to serve as the basis for further analysis ( Figure 1) and distributed as part of publications. These downstream analyses may leverage any of the existing work-flow management tools. Our goal is that data sets forming the basis of scientific findings can be confidently shared in their processed form which is often much smaller and easier to distribute.

Within the VISC, a team of analysts, statistical programmers, and data managers work collaboratively to analyze pre-clinical data arising from multiple trials. There are multiple assays per trial. The challenges associated with ensuring the entire team works from the same version of a frequently changing and dynamic data set, motivated the development of *DataPackageR*. The tool is routinely used to process and standardize trial data for submission to The Collaboration for AIDS Vaccine Discovery (CAVD) Data Space.

We demonstrate how *DataPackageR* is used to process and package multiple types of assay data from an animal trial of an experimental HIV vaccine.

### Data

We demonstrate the use of *DataPackageR* for processing data from a vaccine study, named *MX1*, designed to examine the antibody responses to heterologous N7 Env prime-boost immunization in macaques. The study had four treatment groups plus a control arm, with six animals per group. Samples were collected at three time points: t1: baseline, post-prime 2, post-boost 1, t2: post-boost 2, t3: post-boost 3. Six assays were run at each time point, using either serum samples or peripheral blood mononuclear cells (PBMCs). The assays were: 1) enzyme-linked immunosorbent assay (ELISA), an immunological assay that enables detection of antibodies, antigens, proteins and/or glycoproteins (serum); 2) a neutralizing antibody (Ab) assay (serum); 3) a binding antibody multiplex assay (BAMA) to assess antibody response breadth (serum); 4) a BAMA assay to permit epitope mapping (serum); 5) an antibody dependent cellular cytotoxicity (ADCC) assay (serum); and 6) an intracellular cytokine staining assay to assess cellular responses (PBMCs).

The raw data and environment to reproduce the processing with *DataPackageR* are distributed as a Docker image on hub.docker.com as gfinak/datapackager:latest. We have restricted the number of FCS files distributed in the container to limit the size of the image and speed up processing of FCM data for demonstration purposes.

### Flow cytometry and other assay data

Flow cytometry (FCM) is a high content, high throughput assay for which VISC leverages specialized data processing and analytics tools. Raw FCS files and manual gate information in the form of FlowJo (FlowJo LLC, Ashland, OR) workspace files are uploaded directly to VISC by the labs. The raw data are processed with open source BioConductor software ( *flowWorkspace*) to import and reproduce the manual gating, extract cell subpopulation statistics, and access the single-cell event-level data required for downstream modeling of T-cell polyfunctionality and immunogenicity ^34^,^36^. Tables of extracted cell populations, cell counts, proportions, and fluorescence intensities are included in study packages, together with an Rmarkdown vignette describing the data processing. Due to the size of the raw FCS files, they are imported for processing from a location external to the data package source tree, so that the raw files are not part of the final package, but vignettes outlining the data processing are automatically included.

Remaining assay data are of reasonable size and are provided raw data in tabular (csv) form, imported into the package, processed and standardized from the inst/extdata package directory. Users can run and connect to an Rstudio instance in the container, where code and data to build the *MX1* data package reside.

MX1 is just one study from over 33 prepared by VISC over the past 3 year using the *DataPackageR* infrastructure. Each study consists of three to seven data types, each with different QC and standardization criteria, each produced by different labs, all on a different time line. For each data type, VISC produces QC reports for the labs, statistical analysis plans for each data type, statistical reports and analyses for each data type, and an integrated analysis for manuscripts and publications. The data generally arrive over a time period of several months, and reports are also generated over several months. Different individuals are responsible for the generation of different reports. It is critical that all reports are based off the same data set, and that they are updated when individual data sets are updated. For the MX1 study above, the data package was updated 29 times between 2015 and 2017. The data set version was incremented from 0.1.0 to 0.2.21 over that time frame. Six data sets were added to the package over two years, 17 commits were associated with corrections or updates to data sets (e.g. additional or corrected annotations). Six follow up reports based on the data package were updated each time there was an addition, or correction to the data. DataPackageR helped ensure consistency between the different reports and the data, something that would have been incredibly difficult without this framework.

## Caveats

We note a number of limitations with the existing framework that we hope to address in the future. First, our dependence on git and gtihub to version control data sets means that in the long-term the size of the local copy of a repository will grow with every changed data set. We are exploring approaches to cache older versions of data sets remotely, analogous to approaches taken by BioConductor’s ExperimentHub ( https://doi.org/doi:10.18129/B9.bioc.ExperimentHub), and packages like datastorr ( https://github.com/ropenscilabs/datastorr).

## Summary

Reproducibility is increasingly emphasized for scientific publications. We describe a new utility R package, *DataPackageR* that serves to help automate and track the processing and standardization of diverse data sets into *analysis-ready data packages* that can be easily distributed for analysis and publication. *DataPackageR*, when paired with a version control system such as *git*, decouples data processing from data analysis while tracking changes to data sets, ensuring data objects are documented, and keeping a record of data processing pipelines as vignettes within the data package. The principle behind the tool is that it remains a lightweight and non-intrusive framework that easily plugs into most R-based data analytic work-flows. It places few restrictions on the user code therefore most existing scripts can be ported to use the package. The VISC has been using *DataPackageR* for a number of years to perform reproducible end-to-end analysis of animal trial data, and the package has been used to publicly share sets for a number of published manuscripts ^35^,^37^,^38^.

## Software and data availability

Source code available from: http://github.com/RGLab/DataPackageR

Archived source code as at time of publication: https://doi.org/10.5281/zenodo.1292095 ^39^

Reproducible examples: http://hub.docker.com/r/gfinak/datapackager/

License: MIT license

Partial data for the MX1 study to demonstrate processing using *DataPackageR* are available as a docker container from gfinak/DataPackageR:latest. The processed MX1 data are available on the CAVD DataSpace data sharing and discovery tool at http://dataspace.cavd.org under study identifier, CAVD 451. At the time of publication the complete data set is available to CAVD members only.
