# Supplementary material for: DataPackageR: Reproducible data preprocessing, standardization and sharing using R/Bioconductor for collaborative data analysis
Source: Gates Open Res. 2018 Jul 10;2:31. Originally published 2018 Jun 22. [Version 2] doi: 10.12688/gatesopenres.12832.2 (PMC6139382; doi:10.12688/gatesopenres.12832.2)
Supplement: Peer review history

# Peer review: Referee response for version 2

**DOI:** 10.21956/gatesopenres.13917.r26542

**Published:** 2018-07-25

**Authors:** Ben Marwick

**Affiliations:** 1 Department of Anthropology, University of Washington, Seattle, WA, USA

This is a well-written and clear paper about a very interesting package that helps to raise awareness about the challenges of collaborating on data sets that change over time. The package describes is a useful addition to the toolkit of R-based infrastructure to promote transparent and open research. 

 Previous work includes workflowr and drake. This is a very incomplete list. There are many other R packages that deal specifically with data provenance that would be relevant to note here. And it's not clear specifically what the previous work lacks? The authors say that rerunning rnaseq code is impractical and time consuming, but there are many existing approaches to manage that problem, e.g. the venerable make. It's not clear how this package solves a problem that can't be solved with existing packages. How is their package different to any previous effort? What specific problem does it solve? What makes it notable and a unique contribution? I'm confident the authors can answer these questions, and I think it would make the ms. more useful and impactful if they could do so more directly and specifically. The reader will want to know that this is not just a re-invention of the wheel, or a thin wrapper around something they already use heavily. 

 The detail that it is a lightweight alternative to Galaxy is an important motivation that should be noted to the reader earlier in the paper. This sentence seems to hint at the unique value of this package: "it is designed to reproducibly prepare data for analysis, using an existing code base" but it needs more description and elaboration to more clearly show the reader why this package is necessary. It would also be helpful to hear more about the research context where this package is optimal, you say something about medium-sized organisations. But for researchers who don't do DNA analysis and flow cytometry, a little more detail on the size and structure of the research team and typical research process will help them recognise if this solves a problem they also have. 

 It is a nice touch to prompt documentation of data sets. I wonder about having so many top-level non-standard package files, that will be off-putting to some people who are not used to overloading the package structure that. 

 How do packages made by this package communicate to the user the initial source of the data, and the final location? For example, how does it capture the date, time, instrument, etc where the data came from, and the DOI and repository of where the data has been finally deposited? Is this added in free text in the roxygen documentation, or are there fields especially for this? The system provided by this package seems a bit disconnected from the full pipeline, which might limit its archival value. Seems that it's optimised for work-in-progress data management, with less concern for the long term. Where do the authors recommend the resulting packages go when the work is complete? I don't think GitHub is a good solution for long term archiving. 

 There is a mention of Docker in the 'Use Case' section, but no explanation of whether or not the package no handles this, or not. Also in this section it would be good to have URLs to the packages being discussed so the reader can inspect them in more detail, and see proof of the concept. 

 In the 'caveat' section there is a mention of the size of the data, but no specific details. It would be more helpful to users to know the ballpark sizes that this package is good for. It is up to 5 MB of processed data files? Do the authors have any guidance or rules of thumb such as 10 MB of FCM raw data can typically be stored as 5MB of package data? These would be very helpful for potential users to gauge the usefulness of this tool their own situation. 

 In the console output for DataPackageR::datapackage_skeleton it would be nice to use usethis:::done() for more consistent formatting of the messages. Also, in the example code on the GitHub readme, creating the pkg in a tmp directory makes it quite hard to find to edit the yml file and inspect the contents. I understand that using a tempfile makes it easy to test and knit, but it's not very user-friendly. Perhaps worth noting somewhere that the packages this package creates are not CRAN-ready (I got 1 warning and 1 note with the mtcars20 package). It would be good to have some guidance about how the authors recommend packages created by this method be shared (presumably github?).

I have read this submission. I believe that I have an appropriate level of expertise to confirm that it is of an acceptable scientific standard, however I have significant reservations, as outlined above.

# Peer review: Referee response for version 2

**DOI:** 10.21956/gatesopenres.13917.r26579

**Published:** 2018-07-24

**Authors:** Ted Laderas

**Affiliations:** 1 Department of Medical Informatics and Clinical Epidemiology, Knight Cancer Institute, Oregon Health & Science University (OHSU), Portland, OR, USA

The authors have designed a package that allows for bundling of multiple data types that can be versioned, along with preprocessing scripts and raw data into an R data package. The main use case for this package seems to be systems biology projects where multiple datatypes (such as RNAseq, DNAseq, flow cytometry, etc.) need to be aggregated together for analysis by multiple groups. As a systems biologist, I believe that `DataPackageR` does fill a useful niche between complete research compendia and storage of results in a database, which may require dedicated data modeling.

 I have run the docker container example and built the MR1 data package in the vignette. The ability to version aggregated datasets and tie an analysis to a version is a very important one. As the other reviewer did suggest, putting large datasets into GitHub would cause the repo to become very large and I'm glad that the authors have tried to address this.

 I see `DataPackageR` working well with both workflow tools (such as `drake`) but also potentially data integrity testing tools (such as `assertr`) to guarantee a common version of the data. I believe it serves a useful purpose within data analysis.

I have read this submission. I believe that I have an appropriate level of expertise to confirm that it is of an acceptable scientific standard.

# Peer review: Referee response for version 2

**DOI:** 10.21956/gatesopenres.13917.r26556

**Published:** 2018-07-11

**Authors:** Aaron T.L. Lun

**Affiliations:** 1 Cancer Research UK Cambridge Institute, University of Cambridge, Cambridge, UK

The authors have addressed my concerns in their responses and revisions.

I have read this submission. I believe that I have an appropriate level of expertise to confirm that it is of an acceptable scientific standard.

# Peer review: Referee response for version 1

**DOI:** 10.21956/gatesopenres.13908.r26541

**Published:** 2018-06-25

**Authors:** Aaron T.L. Lun

**Affiliations:** 1 Cancer Research UK Cambridge Institute, University of Cambridge, Cambridge, UK

In this article, Finak et al. describe a new package for pre-processing and sharing of data within the R programming language. Their **DataPackageR** package provides a standardized framework for traceable pre-processing and version control of R data objects, facilitating reproducible research across a diverse team of collaborators. The manuscript is clear and concise, and the function and benefits of the software are clear. Nonetheless, I have some comments (listed below) that the authors might consider to improve both the software and the manuscript.

1. The processed data are stored as *RData* files in the *data/* directory and distributed as part of the constructed data package. These files can become somewhat large (> 100 MB), which in and of itself is not a problem; indeed, having to deal with large data files may not be avoidable in some contexts. However, the text on page 5 and Figure 1 suggest that the user should place the generated data files under version control via Git prior to distribution. With Git, every clone of the package will contain every version of all data files, inflating the size of the repository for download and on disk. This may become prohibitive for practical use (e.g., GitHub forbids uploads of files above a certain size), and feels unnecessary given that only one version of the data will be active at any one time. Perhaps the authors would consider supporting the versioning and acquisition of *RData* files from a separate location, mimicking the behaviour of (or directly using) Bioconductor's **ExperimentHub** where large data files are downloaded and cached locally as needed?
2. The YAML header seems to be limited to R scripts or *Rmd* files for data pre-processing. However, as the authors would appreciate, a lot of pre-processing is performed on the command line, e.g., with aligners and related software. It would be awkward (and involve extra work) to have to wrap these scripts in R/ *Rmd* files in order for them to be executed by **DataPackageR**. To provide a concrete example: I would like **DataPackageR** to directly call my existing Bash scripts for alignment of RNA-seq data, followed by execution of *Rmd* reports for assigning reads to genes to get a count table that is saved as an *RData* object.
3. Does the YAML header permit dependencies between scripts? Say I have a long pre-processing pipeline that is split across multiple scripts for convenience. Does the order of files in the header reflected in the order of execution? And are they executed in the same location, so that intermediate files produced by one script (that are not intended to be included in the final package) can be picked up as inputs to the following script?
4. The MX1 use case on pages 6-7 seems under-described - I don't get an intuition as to why **DataPackageR** was beneficial for this project. It would be demonstrative to have some specific examples of how the version control and change tracking were advantageous in a collaborative environment. For example, how many *DataVersion* bumps were performed throughout the course of the project, and why? Were there any issues arising from changes to the processed data, and how were these resolved?

I have read this submission. I believe that I have an appropriate level of expertise to confirm that it is of an acceptable scientific standard, however I have significant reservations, as outlined above.
